# The monocyte-to-osteoclast transition in rheumatoid arthritis: Recent findings

**DOI:** 10.3389/fimmu.2022.998554

**Published:** 2022-09-12

**Authors:** Naoki Iwamoto, Atsushi Kawakami

**Affiliations:** Department of Immunology and Rheumatology, Division of Advanced Preventive Medical Sciences, Nagasaki University Graduate School of Biomedical Sciences, Nagasaki, Japan

**Keywords:** monocyte, osteoclast, rheumatoid arthritis, disease modifying anti-rheumatic drugs (DMARD), microRNA

## Abstract

Rheumatoid arthritis (RA) is an autoimmune disease characterized by joint inflammation leading to joint destruction and deformity. The crucial role of osteoclasts in the bone erosion in RA has been demonstrated. Deregulated osteoclastogenesis which is affected by environmental factors including the inflammatory state, as well as genetic and epigenetic factors, is one of hallmarks of RA pathogenesis. An enhanced-monocyte-to-osteoclast transition plays an important role in osteoclast upregulation in RA because under specific stimuli, circulating monocytes might migrate to a specific location in the bones and fuse with each other to become mature multinucleated osteoclasts. To understand the mechanism of bone damage in RA and to develop novel treatments targeting osteoclast upregulation, it is important to clarify our understanding of the monocyte-to-osteoclast transition in RA. Several potential targets which inhibit both inflammation and osteoclastogenesis, as well as regulators that affect the monocyte-to-osteoclast transition have been revealed by recent studies. Here, we review the factors affecting osteoclastogenesis in RA, summarize the anti-osteoclastogenic effects of current RA treatments, and identify promising therapeutic targets relating to both inflammation and osteoclastogenesis.

## Introduction

Rheumatoid arthritis is a chronic inflammatory autoimmune disease affecting approximately 1% of the world’s population, caused by multiple genetic, epigenetic and environment factors. RA is systemic disease characterized by chronic arthritis that ultimately results in joint destruction (1). Advanced osteoarticular destruction and deformation can cause irreversible loss of function, in turn leading to physical disabilities that negatively impact quality of life. Therefore, prevention of joint destruction by early diagnosis and early therapeutic intervention is critical in the management of RA (2). Osteoclasts play a central role in bone destruction. Osteoclasts are multinuclear giant cells derived from the monocyte-macrophage lineage and are responsible for bone resorption. Under specific stimuli, circulating monocytes migrate to a specific location in the bones and then fuse with each other to become mature multinucleated osteoclasts (3, 4). Current therapies for RA are not effective in all patients, recently several studies reported that approximately 3~10% of RA patients have been estimated as “difficult-to-treat” RA which defined by multiple biologic disease modifying anti rheumatic drugs (bDMARDs)/targeted synthetic DMARDs failure, rapid progressive bone destruction, moderate disease activity despite appropriate treatment and so on (5–7). Moreover, not all patients can be treated due to the high cost of medication and the occurrence of adverse events such as infection. Therefore, development of novel treatments targeting specific pathogenic cells such as osteoclasts are needed.

Many research groups have investigated how osteoclastogenesis is enhanced in RA or arthritic conditions *in vivo*. Elucidating such dysregulation in RA might help not only reveal the pathogenesis of RA but also point toward new therapeutic targets. Inflammation is closely related to enhanced osteoclastogenesis in RA; however, other osteoclastogenesis-enhancing mechanisms exist in the pathogenesis of RA.

In this review, we focus on the pathological enhancement of the monocyte-to-osteoclast transition in RA mainly independent of cytokines. Additionally, we summarize the osteoclastogenesis-inhibiting effect of current RA treatments and promising therapeutic targets related to both inflammation and osteoclastogenesis in RA.

## Characteristics of monocyte-to-osteoclast transition in RA

Increases in both the number of circulating osteoclast precursor cells/monocyte and in the bone resorption ability of osteoclasts have been reported in RA. Osteoclasts generated from peripheral blood mononuclear cells (PBMCs) including osteoclast precursor cells/monocytes from RA patients showed higher bone resorption compared with those from ankylosing spondylitis patients (8). Mononuclear cells from RA patients with infliximab treatment showed reduction of bone resorption ability. A study conducted by Ikics et al. clearly revealed data showing increased osteoclastogenic potential in RA (9). When similar numbers of PBMCs from RA patients and healthy controls were cultured in osteoclastogenic conditions (cultured under the presence of the receptor activator of nuclear factor-κB ligand (RANKL) with macrophage colony-stimulating factor (M-CSF)), more osteoclasts formed in the RA than the control culture. Spontaneous osteoclast differentiation has also been suspected in RA (10). Hydrochloric acid released by mature osteoclasts close to the ruffled border of osteoclasts solubilizes calcium from the bone matrix and produces matrix metalloproteinases (MMPs) and cathepsin K, which degrade the remaining bone matrix. Mononuclear cells from RA synovial fluid have been shown to express these genes after 21 days’ culture without addition of RANKL, indicating spontaneous osteoclast formation.

As mentioned previously, in RA patients the osteoclastogenic potential of PBMCs is increased; moreover, elevated osteoclast formation in RA has been shown to be correlated with bone loss (11). The number of osteoclasts generated from RA mononuclear cells was correlated with the patients’ sharp score (the joint destruction score evaluated using X-ray) and the lumbar T-score. Also in this experiment, the osteoclast formation of mononuclear cells from RA patients was significantly higher than that from healthy donors. In addition to enhanced osteoclastogenesis, RA affects the longevity of osteoclasts; that is, the number of osteoclasts undergoing apoptosis after osteoclast formation was significantly lower in RA patients than in healthy donors (12).

Joint inflammation contributes to osteoclast-causing bone destruction; thus, in the inflammatory environment of RA, circulating monocytes might continue to transfer to the joint cavity and differentiate into osteoclasts. In a murine model, osteoclasts in the pannus originated from circulating bone marrow-derived cells, not from locally resident macrophages (13). These arthritis-associated osteoclastogenic macrophages (CX_3_CR1^+^HLA-DR^hi^CD11c^+^CD80^−^CD86^+^ cells) are present in the inflamed synovitis and are distinct from conventional osteoclast precursors in homeostatic bone remodeling. Moreover, these arthritis-associated macrophages (CX_3_CR1^+^HLA-DR^hi^CD11c^+^CD80^−^CD86^+^ cells) were detected in synovial samples from human patients with RA.

Classification of monocyte subsets using CD14 and CD16 expression have been a major focus in the analysis of monocyte function. Depending on their expressions of CD14 and CD16, monocytes are defined as classical monocytes (CD14^++^CD16^-^), intermediate monocytes (CD14^++^CD16^+^) and non-classical monocytes (CD14^+^CD16^++^) (14). The expression of CD14 and CD16 is upregulated in the monocytes of RA patients (15, 16); among these three subsets, the intermediate monocytes are dominant in the peripheral blood and synovial tissue of RA (17, 18). Intermediate monocytes secrete pro-inflammatory cytokines such as tumor necrosis factor-α (TNF- α), interleukin-1β (IL-1 β) and IL-6, and they can differentiate into inflammatory macrophages (M1 macrophages) (19, 20). Moreover, the intermediate monocytes in RA are characterized by an increased expression of HLA-DR, and these HLA-DR-positive intermediate monocytes express a high level of costimulatory molecules (CD80 and C86), which promote the induction of IL17^+^CD14^+^ T-cells (17, 21, 22). The relative compositions of classical and non-classical monocytes in RA have been controversial. Some reports have described increased proportions of classical monocytes in RA compared with healthy subjects whereas other reports have shown increased proportions of non-classical monocytes (22, 23). From the view of osteoclastogenesis, the classical monocytes are the most important of the three subsets. Analysis of osteoclastogenic function focusing on CD14/CD16 expression has shown that the monocytes that can differentiate into osteoclasts are CD14-positive but CD16-negative, i.e., CD14^+^CD16^-^ monocytes are the precursors of circulating osteoclasts (24).

## Recent findings of regulators modulating the monocyte-to-osteoclast transition in RA

### Osteoclast-associated receptor

Compared with monocytes from healthy donors, those from the peripheral blood of RA patients showed upregulation of the osteoclast-associated receptor (OSCAR), which is an activating receptor expressed by human myeloid cells, for which both collagen type I (COLI) and collagen type II (COLII) serve as ligands (25, 26). This OSCAR-collagen interaction stimulates RANKL-dependent osteoclastogenesis. Interestingly, OSCAR expression is correlated with disease activity and acute-phase reactant concentrations. OSCAR-collagen interactions are also involved in cytokine production; namely, COL2 stimulates the release of proinflammatory cytokines by monocytes whereas this effect was completely blocked in the presence of OSCAR-antibody.

### M1/M2 monocytes

The M1/M2 subsets of macrophages are well-known for being involved in disease pathogenesis including that of RA. Interestingly, M1-like and M2-like subsets are present in macrophage precursor cells, i.e., monocytes. They were first reported to be associated with diabetes mellitus, then also discovered in hypercholesterolemia (27, 28). M1 monocytes are defined as positive for CD14, CD68, and CCR2, and M2 monocyte are defined as positive for CD14, CX3CR1, and CD163 or CD206. In RA, the M1/M2 ratio of monocytes is correlated with osteoclastogenesis and cytokine production from monocytes (29). Specifically, the number of osteoclasts differentiated from the monocytes of RA patients with high M1/M2 ratios was significantly greater than that from patients whose monocytes had low M1/M2 ratios. M1-dominant monocytes were also found to secrete more IL-6 compared with M2 monocytes.

### Combination of TNF and IL-6 stimulation

Although the presence of RANKL-independent pathway in inflammatory-osteoclastogenesis pathway is controversial because, for example, there is the fact that the TNF-α overexpression failed to induce bone destruction in RANKL-deficient mice (30), Yokota et al. recently reported that PBMC can differentiate into osteoclasts by a combination of TNF and IL-6 stimulation without RANKL (31). The behavior of osteoclasts generated by this combination was different from that of those induced by RANKL. Expression levels of IL-1β, TNF, IL12p40 and MMP-3 were significant increased in the TNF-/IL-6-induced osteoclasts, but not in RANKL-induced osteoclasts. And the joint destruction defined by total sharp score correlated with the number of TNF- and IL-6-induced osteoclasts, but not with the number of RANKL-induced osteoclasts. By contrast, the bone mineral density of the whole body was correlated with RANKL-induced osteoclasts, but not with TNF- or IL-6-induced osteoclasts, indicating that “inflammatory cytokine-generated osteoclasts” may contribute to the pathology of RA but not to whole-body osteoporosis.

### Monocyte-derived dendric cells

Monocyte-derived dendritic cells (Mo-DC), generated from monocytes, were first reported by Steinman et al. on the basis of their unique morphology, which distinguished them from macrophages and dendritic cells, the other source of osteoclast cells in RA (32, 33). The first study conducted by Rivollier et al. demonstrated that Mo-DC, generated by stimulation with granulocyte macrophage-colony stimulating factor (GM-CSF) and IL-4, differentiated into osteoclasts in cultivation with RANKL and M-CSF, and this osteoclastogenesis was greatly enhanced by adding synovial fluid from RA patients (34). After this study, the detailed mechanism of RA synovial fluid-enhanced osteoclastogenesis of Mo-DC was reported (35). The Mo-DC-to-osteoclast transition was correlated with peptidyl arginase deiminase (PAD) activity and protein citrullination. PAD enzymes govern the citrullination process, which is dysregulated in RA and contributes to both the production and maintenance of anti-citrullinated protein antibody (ACPA). Purified ACPA was shown to enhance OC differentiation from Mo-DC, and this enhancement might be caused by targeting citrullinated actin and vimentin deposited on the Mo-DC surface, colocalizing with ACPAs binding to the cells. This study suggested that protein citrullination and ACPA binding to immature DCs might one of the mechanisms of bone erosion in ACPA-positive RA.

### MicroRNAs

MicroRNAs (miRNAs) are evolutionarily conserved small non-coding RNAs (length is 18-25 nucleotides) that regulate gene expression at the post-transcriptional level. There is increasing interest in the involvement of miRNAs in autoimmune diseases including RA. MiRNAs modulates cell proliferation, apoptosis and cell differentiation including both osteogenesis and osteoclastogenesis (36–38). Several miRNAs have been reported in relation with the monocyte-to-osteoclast transition in RA (**Table 1**). Although miR-146a was found to be upregulated in PBMC, synovial fluid and synovial fibroblasts in RA (45), miR-146a behaved as a negative regulator of inflammation (46). Regarding osteoclastogenesis, miR-146a also showed a protective role in inflammatory arthritis: overexpression of miR-146a significantly reduced the osteoclast formation of PBMCs from RA patients, and administration of double-stranded miR-146a prevented joint destruction in collagen-induced arthritis (CIA) mice (47). Interestingly, a recent study revealed that the cells playing a central role in this effect are monocytes: specifically, miR-146a overexpression in LY6C^hi^ monocytes decreased joint destruction in CIA mice (48). MiR-125a has been proposed as an important regulator of the innate immune and inflammatory responses in various inflammatory diseases including RA (49, 50). Plasma levels of miR-125a were found to be elevated in RA, suggesting that this miR might be useful as another biomarker for RA (39, 40). MiR-125a has been founded to be deeply related with monocytes/macrophages, especially macrophage polarization (41, 42). In addition, miR-125a is important for osteoclastogenesis, upregulated during the monocyte-to-osteoclast transitio (41, 43). Overexpression of miR-125a inhibited TNF receptor superfamily member 1B gene (TNFRSF1B) protein expression and promoted osteoclast differentiation whereas inhibition of miR-125a showed an opposite result; furthermore, it was shown that mir-125a promotes osteoclastogenesis by targeting TNFRSF1B (44). MiR-223 has also been thought to be related to osteoclastogenesis in RA although the exact effect on osteoclastogenesis is controversial. One study indicated that miR-223 was highly expressed in the RA synovium, leading to the *in vitro* inhibition of osteoclastogenesis (51) whereas another study indicated miR-223 upregulation in the RA synovium, but knockout rather than overexpression of miR-223 decreased osteoclastogenesis (52). Another possible miR related to osteoclastogenesis in RA is miR-124. Expression of miR-124a was decreased in RA synovial fibroblasts, and in a rat model of arthritis (adjuvant-induced), rats with overexpression of miR-124 (rat analogue of human miR-124a) showed reduced osteoclastogenesis and the expression levels of RANKL, integrin β1 and nuclear factor of activated T cells cytoplasmic 1 (NFATc1) (53, 54).

**Table 1:** Summary of microRNA related to osteoclastogenesis in RA.

microRNA	Targets *	Effect on bone-related cell	Bone-specific findings *in vivo*	Site of expression in RA	Reference
miR-146a	IRF3, TRAF6,IRAK1/2, INHBA	Osteoclastogenesis↓	Prevents joint destruction in CIA mice through monocytes	PBMCs, Synovial fluid Synovial fibroblast	(35–38)
miR-125a	TNFRSF1B	Osteoclastogenesis↑	Not observed	Plasma	(39, 40)
miR-223	Pknox1	Osteoclastogenesis↑/↓	Reduces bone erosion in CIA mice	Synovial fibroblasts	(41, 42)
miR-124	C/EBP-α	Osteoclastogenesis↓	Decreases osteoclastogenesis and RANKL expression in AIA rats	Synovial fibroblasts	(43, 44)

*These targets are not specific to osteoclast.

Bone-related cell means joint-destruction and bone remodeling associated cell such as osteoclast and osteoblast.

IRF3 interferon regulatory factor 3, TRAF6 tumor necrosis factor receptor-associated factor 6, IRAK1/2 interleukin 1 receptor associated kinase 1/2, INHBA inhibin beta A chain, CIA collagen-induced arthritis, PBMC peripheral blood mononuclear cell, TNFRS1B tumor necrosis factor receptor superfamily member 1B, Pknox1 PBK/Knotted 1 homeobox 1, C/EBP-α PCCAAT/enhancer binding protein-α, RANKL receptor activator of nuclear factor-κB ligand, AIA adjuvant-induced arthritis.

## Targeting osteoclastogenesis by current RA treatment

The available disease modifying anti rheumatic drugs (DMARDs) are subdivided into conventional synthetic DMARDs (csDMARDs: methotrexate (MTX), salazosulfapyridine, etc), bDMARDs and targeted synthetic DMARDs. The effect on osteoclastogenesis by these DMARDs have been reported (**Table 2**). In the current treatment strategy, after the diagnosis of RA, MTX is suggested as a first-line therapy of RA (phase1). In case MTX is contraindicated, another csDMARD is used. The patient moves to phase 2 if treatment by phase 1 agents shows inadequate response (2).

**Table 2:** The effect of current RA treatments on bone-related cells.

Drug	Effect on bone-related cells	Bone-specific findings *in vivo*	Reference
MTX	Osteoclastogenesis↓	Improves bone mass by decreasing osteoclast-born resorption in AIA rats	(51, 52)
TNF-inhibitor	No direct effect on osteoclasts Increases conversion of bone-resorbing osteoclasts to non-resorbing cells	Prevents systemic bone mass reduction in CIA mice	(55, 56)
IL-6 inhibitor	No direct effect on osteoclasts Increases conversion of bone-resorbing osteoclasts to non-resorbing cells	Increases apoptosis of osteoclasts in IL-6 knockout mice	(56–58)
CTLA4-Ig	Osteoclastogenesis↓ Promotes polarization of M1to M2 macrophages Inhibits osteoclast precursors from attaching to bone surface	Decreases osteoclastogenesis in TNF-α transgenic mice	(54, 56, 59–61)
Anti-RANKL antibody	Osteoclastogenesis↓	Decreases osteoclastogenesis at the site of joint inflammation in CIA mice	(62–64)
JAK inhibitor	No direct effect on osteoclasts Osteoblasts↑	Promotes osteogenesis in estrogen-deficiency mice and inflammation arthritis mice	(65, 66)

Bone-related cell means joint-destruction and bone remodeling associated cell such as osteoclast and osteoblast.

MTX methotrexate, AIA adjuvant-induced arthritis, TNF tumor necrosis factor, CIA collagen-induced arthritis, IL-6 interleukin 6, CTLA cytotoxic T-lymphocyte antigen, RANKL receptor activator of nuclear factor-κB ligand, JAK janus kinase.

### csDMARDs

The direct effects of MTX on osteoclastogenesis were reported early on. Specifically, Segawa et al. reported that MTX improved bone mass by preventing decreased osteogenesis and increased bone resorption in adjuvant-induced arthritis rats (59). After that, Kanagawa et al. reported that MTX significantly inhibited osteoclastogenesis through inhibiting RANK-dependent calcium influx into osteoclast progenitors (60).

### Biologic DMARDs

bDMARDs are used in phase 2 in the treatment strategy, in case MTX/cs DMARDs do not result in an adequate response. In this group of medications, TNF-inhibitors, IL-6 inhibitors and CTLA4-Ig are widely used for treatment of RA. Protection of joint destruction by suppressing inflammation is the main effect of bDMARDs, but inflammation-independent effects of bDMARDs also exist (61). Among the bDMARDs, the one most deeply explored regarding inhibition of osteoclastogenesis is CTLA4-Ig. The binding of CTLA4-Ig to CD80/86 induced activation of the enzyme indoleamine 2,3-dioxygenase (IDO) in osteoclast precursor cells, leading to degradation of tryptophan and the promotion of apoptosis (55, 67). This direct osteoclastogenesis-inhibitory effect of CTLA4-Ig was further analyzed recently (57). Experiments using mice bone marrow macrophages revealed that CTLA4-Ig directly inhibited osteoclastogenesis by interfering with intracellular calcium oscillations. Moreover, CTLA4-Ig treatment promotes M1-to-M2 macrophage polarization, M2 macrophage play anti-osteoclastogenic role as compared with M1 macrophage (58), on monocyte-derived macrophages from RA patients (56). Regarding TNF inhibitor and IL-6 inhibitor, there is no evidence for direct inhibition of osteoclastogenesis; however, TNF-α inhibition has been shown to decrease systemic bone mass reduction in the collagen-induced arthritis model; in IL-6 knockout mice, osteoclast apoptosis is promoted (62–64). In addition to the effect on osteoclastogenesis, the effects on bone reportion by these 3 classes of bDMARDs were recently revealed by Matsuura et al. using *in vivo* visualization of osteoclast behavior (68). In that study, TNF inhibitor and IL-6 inhibitor affected mature osteoclasts and switched bone-resorbing osteoclasts to non-resorbing cells; on the other hand, CTLA4-Ig had no action on mature osteoclasts but affected osteoclast precursors by decreasing their firm attachment to the bone surface, thereby preventing bone resorption.

### Anti-RANKL antibody

The anti-RANKL antibody denosumab is another biologic approved in Japan to inhibit the progression of bone erosion associated with RA. Because the RANK-RANKL pathway is essential for osteoclastogenesis (69), denosumab, through its selective binding to RANKL, inhibits the RANK-RANKL interaction, inhibiting osteoclastogenesis. Phase 2 and 3 clinical trials revealed that denosumab did not inhibit inflammation of RA, clinically defined as the disease activity score, but did inhibit bone erosion; this might come from the mechanism by which denosumab only inhibits osteoclasts but not other cells that play a critical role in RA inflammation such as synovial cells and macrophages (65, 66).

### JAK inhibitors

Small molecules are playing an increasing role in the treatment of RA (70); in particular, JAK inhibitors are small-molecule drugs that inhibit the JAK-STAT pathway activity, which is involved in many biologic functions including the activation of immune cells and is associated with several cytokines that are closely related to the pathogenesis of RA (71).*In vitro* studies have revealed that JAK inhibitors have no direct effects on osteoclasts, but they do suppress osteoclastogenesis *via* inhibiting RANKL expression on mesenchymal cells such as osteoblasts (72, 73). Interestingly, JAK inhibitors promote osteogenesis (72). In experiments using two different bone-loss model mice (estrogen-deficiency and inflammation arthritis model), the JAK inhibitors tofacitinib and baricitinib showed no direct effects on osteoclasts, but the miRNA sequencing and ingenuity pathway analysis of osteoblasts showed robust up-regulation of Wnt1 and β-catenin; i.e., JAK inhibitors promote osteogenesis by activation of the canonical Wnt signaling pathway.

## Future therapeutic perspective targeting osteoclastogenesis in RA

Research is ongoing into inhibiting osteoclastogenesis as a treatment option for RA (**Table 3**). One such candidate is the targeting C-X3-C motif chemokine ligand 1 (CX3CL1)- C-X3-C motif chemokine receptor 1 (CX3CR1) axis. CX3CR1, a unique receptor for CX3CL1, is expressed in monocytes/macrophages, dendritic cells and osteoclast precursor cells (86–89). In RA synovial tissue, the expression of CX3CL1 is high and the serum level of soluble CX3CL1 correlates with the disease activity of RA (74, 90). As mentioned above, CX3CL1 plays an important role in the pathogenesis of RA, and a clinical trial using an anti-CX3CL1 monoclonal antibody have been done (75–77). Since CX3CL1 promotes osteoclastogenesis, anti-CX3CL1 therapy has the potential to prevent joint destruction by targeting osteoclast in RA (86).

**Table 3:** Potential therapeutic targets for treatment of RA by targeting osteoclastogenesis.

Molecule	Effect on bone-related cells	Effect on inflammation	Reference
CX3CL1	Osteoclastogenesis↑	Activation	(70)
GM-CSF	Osteoclastogenesis↑	Activation	(74–77)
Sema3A	Osteoclastogenesis↓	Suppression	(78–80)
Sema4D	Osteoblasts↓	Activation	(81, 82)
CaMK4	Osteoclastogenesis↑	Activation	(83)
Notch	Osteoclastogenesis↑ Osteoblasts↓	Activation	(84, 85)

Bone-related cell means joint-destruction and bone remodeling associated cell such as osteoclast and osteoblast.

CX3CL1 C-X3-C motif chemokine ligand 1ethotrexate, GM-CSF granulocyte macrophage-colony stimulating factor, Sema3A semaphoring 3A, Sema4D Semaphorin 4D, CaMK4 calcium/calmodulin-dependent protein kinase IV.

The contribution of GM-CSF to the pathogenesis of RA, for example, its elevation in synovial effusion, its genetic level and its crucial role in experimental inflammatory arthritis models, have been well documented by several reports (78, 91–94). GM-CSF induces fusion of prefusion osteoclasts to form bone-resorbing osteoclasts and thereby promotes bone erosion (79). Based on these findings, a GM-CSF inhibitor is now being investigated in a clinical trial (80–82). Molecules that inhibit osteoclastogenesis as well as inflammation are a promising therapeutic option for RA. Semaphorin 3A (Sema3A) has been recently identified as an essential player in bone homeostasis, and Sema3A suppresses osteoclastogenesis and increases osteoblastic bone formation (83, 95). Sema3A levels are decreased in RA serum as well as synovial fluid, and administration of sema3A was shown not only to ameliorate inflammation and bone erosion but also to increase bone formation in a serum transfer-induced arthritis model, which indicated that sema3A is both an immunosuppressive factor and osteoprotective factor (96). Semaphorin 4D (Sema4D) is also known to be an osteoimmune molecule in the semaphorin family. In contrast to the effect of Sema3A, Sema4D promotes inflammation and inhibits osteoblastic bone formation (97, 98). Sema4D levels are increased in both RA serum and RA synovial fluid. In a CIA model, treatment with anti-Sema4D antibody suppressed arthritis and reduced proinflammatory cytokine production (98).

Recently, Koga et al. reported the role of calcium/calmodulin-dependent protein kinase IV (CaMK4) in bone destruction under arthritic conditions (84). CaMK4-deficient mice subjected to CIA showed improved arthritis and decreased Th17 cells. This dual effect on Th17 cells and joint destruction was confirmed in cells from RA patients. CaMK4 inhibition suppressed IL-17 production by CD4+ cells from RA patients and suppressed osteoclastogenesis of monocytes from RA patients.

Notch signaling has an important role in RA, Specifically, Notch signal expression and activation can stimulate synovial cells, thereby accelerating the production of proinflammatory cytokines in RA, and Notch signal are highly expressed in RA synovial tissues related to proliferation and invasion of synovial fibroblasts (85, 99–101). The Notch signaling pathway also can affect osteoclastic differentiation and bone balance; thus, Notch inhibition increases bone volume by enhancing osteoblastic bone formation (102, 103).

## Concluding remarks

**Figure 1:**
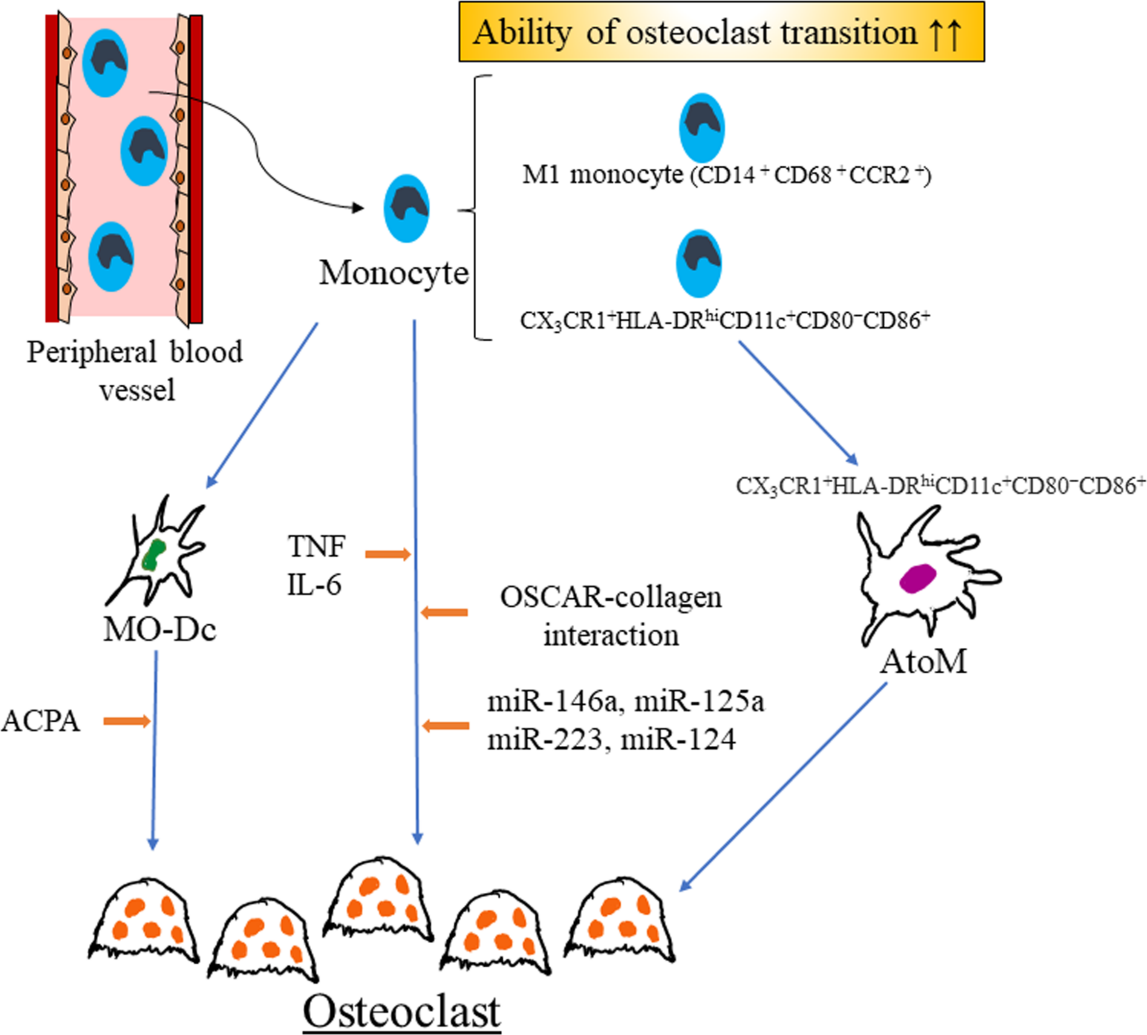
Recent findings of regulators of monocyte-to-osteoclast transition in rheumatoid arthritis. M1 monocyte might have enhanced ability of osteoclast transition as compared with M2 monocyte. Several microRNAs, combination of cytokines and OSCAR-collagen interaction promote osteoclast differentiation in RA. Mo-DCs are generated from monocytes and ACPA enhance osteoclastogenesis of Mo-DCs. In rheumatoid arthritis, arthritis-associated osteoclastogenic macrophages (AtoM) are generated from CX_3_CR1^+^HLA-DR^hi^CD11c^+^CD80^−^CD86^+^ monocyte and differentiated into osteoclast. *RA* rheumatoid arthritis, *Mo-Dc* monocyte-derived dendritic cells*, MO-Dc* monocyte-derived dendritic cells*, ACPA* anti-citrullinated protein antibody, *TNF* tumor necrosis factor, *IL-6* interleukin-6, *OSCAR* osteoclast-associated receptor, *AtoM* arthritis-associated osteoclastogenic macrophages.

Abnormal upregulation of osteoclastogenesis is an important part of the pathogenesis of RA. The monocyte-to-osteoclast transition is crucial for joint destruction although osteoclast precursor cells other than monocytes exist, and osteogenic dysregulation is also important for bone destruction. Numerous studies have shown the environmental, genetic and epigenetic factors that combine to upregulate osteoclastogenesis and modulate osteoclast precursor cells in RA; recent findings regarding these factors such as microRNAs are presented in this review (**Figure 1**). Many other rheumatic diseases such as Sjogren syndrome, systemic lupus erythematosus and inflammatory myositis cause arthritis but no destructive arthritis. Several parts of pathological condition such as abundant production of cytokines, activation of immune cells share with RA, while the enhancing of monocyte-to-osteoclast transition in these rheumatic disease have been rarely reported. Considering these, abnormal monocyte priming by miRNAs, cytokines and so on, monocyte characteristic property such as M1/M2 imbalance might contribute to joint destruction in RA as combined effect. And, though it should be interpreted with caution because *in vitro/vivo* effects of anti-osteoclastogenesis do not always reflect clinical effects of ant-bone destruction in RA, recent studies have revealed potential targets contributing to inhibition of both inflammation and osteoclastogenesis. Although much work remains to be performed to gain an integrated overview of osteoclastogenesis in RA, such research may lead to the discovery of novel therapeutic approaches to RA.

## Author contributions

NI: wrote the manuscript. NI and AK: reviewed and edited the manuscript. Both authors contributed to the article and approved the submitted version.

## Conflict of interest

The authors declare that the research was conducted in the absence of any commercial or financial relationships that could be construed as a potential conflict of interest.

## Publisher’s note

All claims expressed in this article are solely those of the authors and do not necessarily represent those of their affiliated organizations, or those of the publisher, the editors and the reviewers. Any product that may be evaluated in this article, or claim that may be made by its manufacturer, is not guaranteed or endorsed by the publisher.
